# Recovery of Functional Independence After Traumatic Transtentorial Herniation With Duret Hemorrhages

**DOI:** 10.3389/fneur.2019.01077

**Published:** 2019-10-09

**Authors:** Brian L. Edlow, Zachary D. Threlkeld, Katie P. Fehnel, Yelena G. Bodien

**Affiliations:** ^1^Department of Neurology, Center for Neurotechnology and Neurorecovery, Massachusetts General Hospital, Harvard Medical School, Boston, MA, United States; ^2^Athinoula A. Martinos Center for Biomedical Imaging, Massachusetts General Hospital, Charlestown, MA, United States; ^3^Department of Neurology and Neurological Sciences, Stanford School of Medicine, Stanford, CA, United States; ^4^Department of Neurosurgery, Harvard Medical School, Boston Children's Hospital, Boston, MA, United States; ^5^Department of Physical Medicine and Rehabilitation, Spaulding Rehabilitation Hospital, Boston, MA, United States

**Keywords:** Duret hemorrhage, coma, traumatic brain injury, brainstem, herniation

## Abstract

Historically, Duret hemorrhages have conferred a devastating prognosis. However, recent case reports suggest that cognitive and functional recovery are possible after Duret hemorrhages. Here, we describe a patient who recovered consciousness, communication, and functional independence after Duret hemorrhages caused by traumatic transtentorial herniation. We performed prospective, standardized behavioral assessments, structural MRI scans and stimulus-based functional MRI (fMRI) scans during the first 2 years of recovery. The multimodal assessments revealed reintegration of neural networks mediating language and consciousness, concurrent with the reemergence of functional independence. These observations provide insights into network-based mechanisms of recovery from coma and add to a growing body of evidence indicating that Duret hemorrhages are not invariably associated with a poor prognosis.

## Introduction

Historically, Duret hemorrhages have conferred a devastating prognosis (1). Since Henri Duret's 1878 description of brainstem hemorrhages caused by transtentorial herniation (2), Duret hemorrhages have frequently been associated with death, prolonged disorders of consciousness, or devastating functional disability (3–6). However, recent case reports suggest that cognitive and functional recovery are possible after Duret hemorrhages (7–14), including in patients with traumatic brain injury (TBI) (12–14). These reports raise the possibility that historical perceptions about the devastating prognostic implications of Duret hemorrhages may be related to a self-fulfilling prophecy, whereby a radiologic predictor is linked to a poor outcome due to withdrawal of life-sustaining therapy, even if no biological mechanism for such an association exists (15, 16).

Here, we describe a patient who recovered consciousness, communication, and functional independence after Duret hemorrhages caused by traumatic transtentorial herniation. To investigate potential mechanisms of recovery, we enrolled the patient in a prospective longitudinal research study and performed serial, standardized behavioral assessments, structural MRI scans, and stimulus-based functional MRI (fMRI) scans. These multimodal assessments revealed reemergence of functional independence, concurrent with reintegration of neural networks mediating language function.

## Case Report

A healthy 26-year-old left-handed man fell from an 18-foot roof with head strike. Witnesses reported a brief loss of consciousness. Initial neurological examination performed by Emergency Medical Services personnel revealed a Glasgow Coma Scale (GCS) score of 15. The patient was transported to the nearest Emergency Department, where a non-contrast head computed tomography (CT) scan revealed a left temporal bone fracture and a 6 mm left-sided subdural hematoma with 5 mm of midline shift at the septum pellucidum. Multiple facial fractures were also identified, requiring closed reduction of the mandible with placement of arch bars and wires. While in the hospital, he developed severe headaches that were associated with photophobia, phonophobia and a sensation of “whooshing” in the left ear, which was temporally correlated with his pulse. He was discharged home on post-trauma day 8 with no cognitive or sensorimotor deficits.

Headaches, photophobia, phonophobia, nausea, and emesis continued post-discharge, for which he was treated with compazine and dexamethasone. On post-trauma day 14, he experienced acute onset of thunderclap headache while lying in bed, followed by a sudden neurological decline. He called out to his wife that “something is happening with my head,” and seconds later he slumped over and became unresponsive. There was no recent physical activity or exertion, and there were no traumatic events since the 18-foot fall. Upon arrival to the Emergency Department, his GCS score was 4 (Eyes = 1, Motor = 2, Verbal = 1) with a dilated left pupil (size not documented) and absent pupillary responses. Head CT scan showed enlargement of the left-sided subdural hematoma to 14 mm in maximal diameter, causing transtentorial herniation with effacement of the basal cisterns, 2.1 cm of midline shift at the septum pellucidum, and 9 mm of midline shift at the pineal gland. The density characteristics (i.e., Hounsfield units) of the subdural blood suggested mixed acute and chronic components. In addition, a new intraparenchymal hemorrhage within the left temporal lobe was identified, with a volume of approximately 28 mL. He was treated with 100 grams of mannitol and hyperventilation en route to the operating room, where he underwent an emergent left hemicraniectomy for subdural evacuation and placement of left external ventricular drain. A potential arterial source of bleeding, the middle meningeal artery, was identified intraoperatively and cauterized, suggesting a traumatic dural arteriovenous fistula.

**Figure 1:**
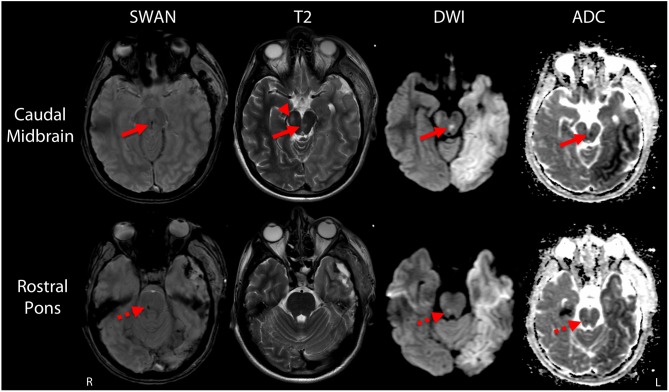
Duret hemorrhages due to traumatic transtentorial herniation. Representative axial images are shown at the levels of the caudal midbrain (top row) and rostral pons (bottom row). In the caudal midbrain, a Duret hemorrhage is indicated by curvilinear hypointense signal at the midline on the susceptibility-weighted angiography (SWAN) image, which corresponds with a hyperintensity on the T2-weighted image (T2) and diffusion-weighted image (DWI), as well as a hypointensity on the apparent diffusion coefficient (ADC) map (solid arrows). A second hypointense lesion is seen in the rostral pons on the SWAN image. The signal properties of this second lesion are similar on the diffusion-weighted image and apparent diffusion coefficient map (dashed arrows), but no corresponding hyperintensity is seen on the T2-weighted image. A T2-hyperintense lesion within the right cerebral peduncle (arrow head) at the level of the caudal midbrain likely represents injury to the descending corticospinal tract fibers due to compression against the tentorium during the herniation event.

**Figure 2:**
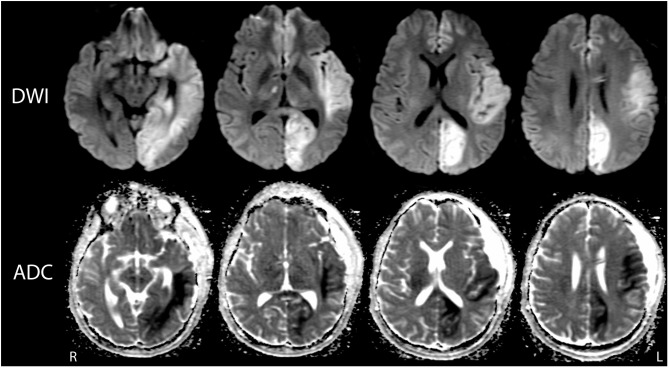
Multifocal infarctions due to vascular compression during transtentorial herniation. Representative diffusion-weighted images (DWI; top row) and apparent diffusion coefficient (ADC; bottom row) images demonstrate ischemic infarctions with the anterior cerebral artery, middle cerebral artery, and posterior cerebral artery territories. The infarctions are indicated by hyperintense signal on DWI and corresponding hypointense signal on ADC. At the time of this MRI scan (post-herniation day 6), the patient's behavioral assessment indicated a minimally conscious state.

Post-operatively, serial head CTs demonstrated resolution of transtentorial herniation and improvement in mass effect. However, multiple large hypodensities were seen in the left hemisphere, suggesting infarctions within the left anterior cerebral artery (ACA), middle cerebral artery (MCA), and posterior cerebral artery (PCA) territories. There was no cerebrovascular stenosis, occlusion, vasospasm, or dissection on CT angiography, and thus, despite the rarity of herniation-related MCA infarction, the infarctions were interpreted as sequelae of vascular compression during herniation. The patient was extubated on post-herniation day 2 and was following simple commands by day 3, although he remained mute. On day 6, he underwent a brain MRI scan. The susceptibility-weighted angiography (SWAN) sequence demonstrated hemorrhages in the caudal midbrain and rostral pons that had not been detected by prior head CT scans (Figure 1). These brainstem hemorrhages were interpreted as Duret hemorrhages, for reasons detailed in the discussion. The diffusion-weighted imaging and apparent diffusion coefficient data confirmed acute infarcts in the left ACA, MCA, and PCA territories (Figure 2). Hyperintense signal on the T2-weighted and diffusion-weighted images was seen within the right cerebral peduncle, likely due to compression of the peduncle against the tentorium during the herniation event. This interpretation is consistent with the left-sided hemiparesis that the patient developed after herniation, known as the “Kernohan's notch” phenomenon (17).

The patient was transferred to the Neurosciences Intensive Care Unit (NeuroICU) at our institution on post-herniation day 9, at which time his GCS score was 9 (Eyes = 3, Motor = 5, Verbal = 1). His pupils were now briskly reactive to light. Intracranial pressure was normal, and his external ventricular drain was removed on day 11. Bedside examinations between post-herniation days 9 and 13 were notable for fluctuating arousal, inconsistent command following, and absence of expressive language, suggesting a minimally conscious state. At the time of the investigational MRI on post-herniation day 14, a comprehensive behavioral examination with the Coma Recovery Scale-Revised (CRS-R) (18) revealed functional object use, indicating recovery to the post-traumatic confusional state (PTCS) (19).

On day 15, the patient was transferred from our NeuroICU to a long-term acute care hospital, where he continued to recover in all cognitive and functional domains. Serial Disability Rating Scale (DRS) (20) scores indicated functional improvement from extreme severe disability to moderate disability. He remained disoriented on consecutive administrations of the Orientation Log, though these assessments may have been confounded by an ongoing language impairment. Two months after the herniation event, he underwent cranioplasty with an unremarkable post-operative CT head within 24 h of cranioplasty, as per institutional protocol, without any evidence of hemorrhage. Three days following cranioplasty (~2 months post-injury), the patient had an acute neurologic decompensation while awaiting discharge. He was found to have an acute epidural and subgaleal hematoma for which he was taken emergently for surgical evacuation. There was no discrete source of bleeding identified. Given this second unexpected bleeding event and generalized oozing at the time of hematoma evacuation, hematology consultation was sought. Comprehensive laboratory evaluation did not reveal a bleeding diathesis. This hospitalization was also notable for multiple generalized tonic-clonic seizures, for which he was treated with anti-epileptic drug therapy. After a ten-day acute hospitalization, he was transferred to an inpatient rehabilitation hospital where he received intensive, multidisciplinary therapy. Additional details regarding behavioral and functional recovery are provided in the Results section below and in Table 1.

**Table 1:** Cognitive, behavioral, and functional assessments.

	**Days post-TBI**	**Days post-herniation**						
	**0–8**	**0–9**	**14**	**15–51**	**53–101**	**175**	**415**	**730**
Event	Admission through discharge after initial fall	Readmission through transfer to NeuroICU	Study participation	LTACH admission through discharge	IRF admission through discharge	Clinical and research follow-up	Clinical follow-up	Clinical follow-up
Diagnosis	No cognitive deficits	Coma-VS/MCS	PTCS	PTCS	PTCS- recovered from PTCS	Recovered from PTCS	Recovered from PTCS	Recovered from PTCS
Level of consciousness/ cognition	GCS = 15	GCS = 4 (E1M2V1); GCS = 9 (E2M6V1)	GCS = 11 (E4M6V1); CRSR = 18 (A3Vi4M602C1 Ar2)	CRSR = 13–23	Cognition could not be validly assessed due to aphasia	Oriented, unable to count from 20 to 1, or recite the months. CAP = 3 symptoms-not confused; TMT A: severely impaired TMT B: unable to complete	Not formally assessed	Brief test of Adult cognition by telephone: impaired abstract reasoning and processing speed
DRS	5	28–23	21	18–6	6	6	5	5
GOSE	5	3	3	3	3	3	5	5
Subjective observations	Headache, nausea, peripheral injuries	Fluctuating arousal, inconsistent command following, no expressive language	Reproducible command- following, expresses himself via writing	Non-fluent aphasia; cognitive- linguistic deficits in attention, recall, problem solving, and executive functioning	Requires 24-h supervision; speaking in short sentences; ongoing impairments in comprehension, expression, and cognition	Near fluent speech, short- term memory impairment, states that thoughts remain fast, but has difficulty expressing himself	Ongoing difficulty with memory (i.e., occasionally misses doses of medications)	Starting to complete tasks that resembled his prior career as a carpenter; ongoing difficulties with reasoning, processing speed, attention

*Timeline of behavioral recovery: A, auditory; Ar, arousal; C, communication; CRSR, Coma Recovery Scale-Revised; CAP, Confusion Assessment Protocol; DRS, Disability Rating Scale; E, eyes; GCS, Glasgow Coma Scale; GOSE, Glasgow Outcome Scale-Extended; IRF, inpatient rehabilitation facility; LTACH, Long Term Acute Care Hospital; M, motor; MCS, minimally conscious state; 0, oromotor; PTCS, post-traumatic confusional state; TBI, traumatic brian injury; TMT, trail making test; V, verbal; Vi, visual; VS, vegetative state*.

## Methods

While in the NeuroICU, the patient's wife provided written informed consent to enroll him in a prospective observational study that included serial behavioral, functional outcome, structural MRI, and stimulus-based fMRI assessments.

### Behavioral and Functional Outcome Assessments

Behavioral assessments utilized the CRS-R, a validated measure of arousal, communication, auditory, visual, motor and oromotor function for patients with impaired consciousness (18); the Confusion Assessment Protocol (CAP), a multidimensional tool that assesses cognition, orientation, and clinical symptoms after TBI (21); the DRS, a measure of global function designed specifically for patients recovering from TBI resulting in coma (20); the Glasgow Outcome Scale-Extended (GOSE), a global measure of functional outcome (22); the Trail Making Test, an assessment of visual attention and task switching (23); and the Brief Test of Adult Cognition by Telephone (BTACT), a telephone-based battery assessing episodic memory, working memory, reasoning, verbal fluency, and executive function (24).

### Structural and Functional MRI Assessments

Methodologic details regarding data acquisition, processing, and analysis have been previously described for the structural MRI (25–27) and stimulus-based fMRI assessments (28, 29). Briefly, on post-herniation day 14 the structural and stimulus-based fMRI scans were performed on a 3 Tesla Siemens Skyra MRI scanner located in the NeuroICU using a 32-channel head coil. For the structural imaging analysis, Duret hemorrhages were mapped on the Harvard Ascending Arousal Network atlas (www.martinos.org/resources/aan-atlas) to determine their degree of overlap with brainstem arousal nuclei that modulate consciousness. Lesion borders were defined by the hypointense signal on the SWAN images (Figure 1). For the stimulus-based fMRI analysis, we utilized a block design with alternating 24 s blocks of language and rest. The language stimulus was a clip from John F. Kennedy's Inaugural Address, which was provided via earphones connected to the scanner console. FMRI analyses were performed using FSL tools (https://fsl.fmrib.ox.ac.uk), with a conservative statistical threshold (*Z* ≥ 3.1) for cluster-corrected changes in the blood-oxygen-level dependent (BOLD) signal.

## Results

### Behavioral and Functional Outcome Measures

Four months after the herniation event, the patient was speaking spontaneously in short sentences with some semantic errors and word-finding difficulties. He had ongoing mild impairment in auditory comprehension and moderate impairment in verbal expression, although he was completely intelligible in the context of performing functional tasks. He was discharged home with 24-h supervision and in-home therapy.

Six months after the herniation event, assessment with the CAP indicated resolution of PTCS but ongoing cognitive impairment, restlessness, and symptom fluctuation. The CAP assessment was no longer confounded by language impairment. He completed Part A of the Trail Making Test in 76 s but was unable to finish Part B, suggesting significant impairment in visual attention and task-switching. He continued to require 24-h supervision at home. His GOSE score was 3, indicating severe lower disability, and his DRS score remained 6. Despite ongoing difficulties with verbal expression, he reported that he could think quickly and fluently, and could sometimes spell or write a word faster than saying it. He was also able to use his phone to text message and express emotions, though with difficulty. The patient and his wife reported that they both noticed improvements on a weekly basis.

At 14 months post-herniation, his GOSE score improved to 5, indicating lower moderate disability, and his DRS score improved to 5, which is in the moderate disability range. He was functionally independent in the home and could complete all activities of daily living without supervision. He still required reminders from his wife to take medications and was unable to return to competitive employment. The patient and his wife agreed that, while there were substantial improvements in language expression and functional independence, memory was an ongoing concern and a barrier to returning to gainful employment. He continued to participate in multidisciplinary therapy three times per week.

At 19 months post-herniation, his GOSE score remained 5, and the DRS score remained 5. Ongoing deficits included right-sided homonymous hemianopia that interfered with functioning in a complex environment, and fatigue, particularly in cognitively challenging situations. He was not yet gainfully employed. At 24 months, GOSE and DRS scores were unchanged. On assessment of basic cognitive functioning using the BTACT (24), he performed below average but within the norm (i.e., within 1.5 standard deviations of the mean, adjusted for age, gender, and education) on tests of basic verbal and working memory, semantic fluency, and executive functioning, but was impaired on assessments of abstract reasoning and processing speed. BTACT scoring was based on the sample means and standard deviations acquired from the Midlife in the United States Refresher Sample (https://www.icpsr.umich.edu/icpsrweb/NACDA/studies/36532/summary). He was still unable to be competitively employed but was beginning to complete tasks that resembled his prior career as a carpenter.

### Structural and Functional MRI

**Figure 3:**
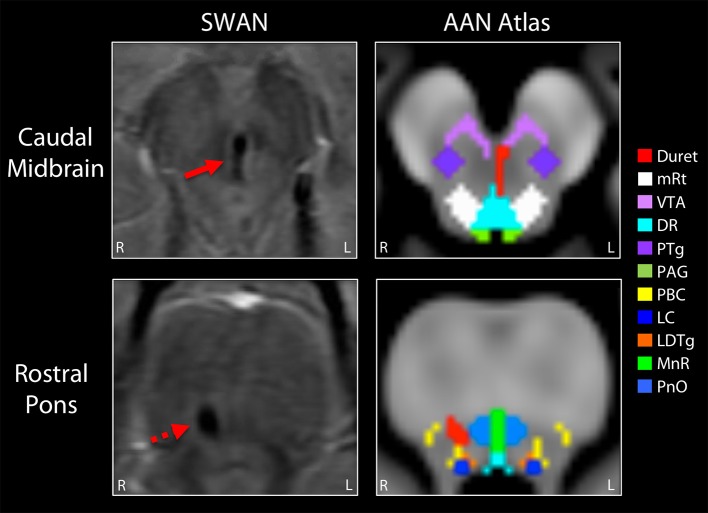
Mapping the Duret hemorrhage lesions to the ascending arousal network atlas. The Duret hemorrhages were manually traced on the Harvard Ascending Arousal Network (AAN) Atlas (www.martinos.org/resources/aan-atlas). Hypointense lesions seen in the caudal midbrain and rostral pons on the susceptibility-weighted angiography (SWAN) images (solid red arrow and dashed red arrow, respectively), were traced in red on the atlas template. These hemorrhages overlapped the dorsal raphe (DR) nucleus and ventral tegmental area (VTA) in the caudal midbrain, as well as the right parabrachial complex (PBC) in the rostral pons. All other AAN nuclei were spared: mRt, mesencephalic reticular formation; PTg, pedunculotegmental nucleus; PAG, periaqueductal gray; LC, locus coeruleus; LDTg, laterodorsal tegmental nucleus; MnR, median raphe; PnO, pontis oralis.

**Figure 4:**
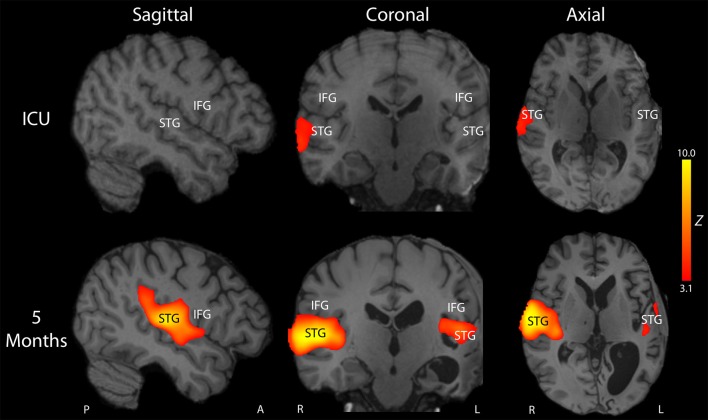
Longitudinal reorganization of the language network after traumatic transtentorial herniation. Stimulus-based functional MRI (fMRI) was performed on post-herniation day 14 in the intensive care unit (ICU; top row) and at 5-month follow up (bottom row). Representative fMRI activation Z-score maps are shown from a sagittal, coronal, and axial perspective, superimposed upon the patient's T1-weighted images. At the time of the ICU fMRI scan, the patient's behavioral assessment indicated that he had recovered to a post-traumatic confusional state, but he remained mute and was unable to consistently follow commands. FMRI revealed a small region of activation in response to spoken language within the right superior temporal gyrus (STG). At 5-month follow up, he was consistently following commands, oriented, and with near fluent language. Repeat stimulus-based fMRI using the same spoken language stimulus demonstrated a larger region of activation in the right STG, as well as new regions of activation within the left STG and right inferior frontal gyrus (IFG). The T1-weighted images demonstrated encephalomalacia of the left hemisphere, with ex vacuo dilatation of the left lateral ventricle. Given that the patient is left-handed and that he did not undergo fMRI before his injury, it is unknown whether he was right-hemisphere dominant or left-hemisphere dominant for language before the injury. Nevertheless, these longitudinal fMRI data indicate that his language network underwent recovery and/or reorganization after the injury and was right-hemisphere dominant post-injury. A, Anterior, L, Left, P, Posterior, R, Right.

On post-herniation day 14, structural MRI revealed that the Duret hemorrhages overlapped the dorsal raphe nucleus and ventral tegmental area in the caudal midbrain, as well as the right parabrachial complex in the rostral pons (Figure 3). All other brainstem nuclei of the ascending arousal network were spared. fMRI acquired during presentation of spoken language revealed a significant increase in the BOLD signal within the right superior temporal gyrus during the language stimulus as compared to rest (Figure 4). A repeat stimulus-based fMRI scan at 5 months showed a larger region of language-induced activation within the right superior temporal gyrus, as well as new areas of language activation within the left superior temporal gyrus and right inferior frontal gyrus (Figure 4).

## Discussion

We report the recovery of consciousness, communication and functional independence in a young man with Duret hemorrhages from traumatic transtentorial herniation. This case adds to a growing body of literature suggesting that Duret hemorrhages are not invariably associated with a poor prognosis. Despite decades of clinical reports describing patients with Duret hemorrhages who either died or survived with devastating cognitive and functional disability, this patient experienced behavioral recovery concurrent with neural network recovery and reorganization. Our findings thus raise the possibility that withdrawal of life-sustaining therapy in prior studies may have led to a self-fulfilling prophecy whereby Duret hemorrhages were erroneously linked with an invariably poor prognosis. Importantly, this single case report does not demonstrate that recovery from Duret hemorrhage is *likely*, but rather that recovery is *possible*.

A key diagnostic consideration in any patient with a brainstem hemorrhage related to TBI is whether the hemorrhage was caused by traumatic axonal injury (TAI) rather than Duret hemorrhage. In this case, several clinical and radiologic observations argue against hemorrhagic TAI as the etiology of the brainstem hemorrhages. First, there was only a brief loss of consciousness at the time of the 18-foot fall. The comatose state did not develop until the subsequent herniation event. Given the strong association between brainstem lesions and loss of consciousness (30, 31), this sequence of events suggests that the brainstem hemorrhages were not primary lesions from the fall, but rather secondary lesions from herniation. Second, the hemorrhage morphology supports the diagnosis of secondary Duret hemorrhage as opposed to primary hemorrhagic TAI lesions. Although there is overlap in morphology between these pathophysiologic entities, Duret hemorrhages tend to be linear or curvilinear (Figure 3) (32), whereas hemorrhagic TAI lesions tend to be ovoid (27, 32). Third, the neuroanatomic location of Duret hemorrhages tends to be localized to the midline (4, 32), whereas hemorrhagic TAI lesions are often eccentrically located in the dorsolateral quadrants of the pontomesencephalic tegmentum (27, 32, 33).

These morphologic and neuroanatomic characteristics of Duret hemorrhages are believed to be related to their pathophysiological mechanism, because downward or lateral displacement of the brainstem causes rupture of intraparenchymal arterioles and/or venules (4, 32), particularly at the level of the caudal midbrain and rostral pons (34). Finally, hemorrhagic TAI in the brainstem does not exist in isolation (33). Rather, if biomechanical forces are severe enough to tear blood vessels and axons deep within the brainstem, these same forces must concurrently tear vessels and axons in the cerebral hemispheres and the corpus callosum (35). Our patient did not have evidence of hemispheric or callosal TAI on multiple MRI scans, providing strong evidence that the brainstem lesions were Duret hemorrhages related to transtentorial herniation.

The structural and stimulus-based fMRI results provide several insights into the potential mechanisms of recovery that enabled our patient to achieve functional independence. Lesion overlap analysis revealed that recovery of arousal (i.e., wakefulness) may be related to sparing of multiple arousal nuclei within the brainstem's ascending arousal network (25). Longitudinal stimulus-based fMRI analysis indicated that the patient's language network recovered and reorganized alongside clinical recovery. Other contributing factors to the patient's recovery likely include the rapid operative reversal of brainstem compression (36), his young age (36), and his strong family support network (37). While the contribution of intensive rehabilitation to recovery from severe TBI, and particularly traumatic Duret hemorrhages, is unknown, it is possible that the patient benefited from extensive inpatient rehabilitation, careful serial assessment, and a multidisciplinary approach to treatment (38). Indeed, in prior rare reports of recovery from Duret hemorrhages caused by traumatic transtentorial herniation, the patients received multidisciplinary rehabilitative care after discharge from the ICU (12–14). It is also notable that the Duret hemorrhages were not detected by CT. It is therefore possible that Duret hemorrhages seen only on MRI carry a more favorable prognosis than those detected by CT. This potential prognostic distinction will require further study, but a similar prognostic phenomenon relating to “CT-negative, MRI-positive” brainstem lesions is increasingly being described in patients with brainstem hemorrhagic TAI (26, 27, 39).

In summary, clinicians should discuss the possibility of recovery from Duret hemorrhages when communicating with families and other surrogate decision-makers in the ICU about goals of care. Given decades of evidence suggesting a high rate of mortality and disability, most patients may never recover functional independence even if aggressive life-sustaining therapy and intensive rehabilitative care are provided. However, mortality rates must be carefully interpreted in the context of self-fulfilling prophecy, whereby mortality is realized “by virtue of having been predicted” (16). This report provides evidence to counter the clinical nihilism that generates self-fulfilling prophecy and provides a rationale for larger studies examining predictors of recovery in patients with Duret hemorrhages.

## Data Availability Statement

The datasets generated for this study are available on request to the corresponding author.

## Ethics Statement

This study involving a human participant was reviewed and approved by the Partners Healthcare Institutional Review Board. A surrogate for the patient/participant provided written informed consent to participate in this study. Written informed consent was obtained from the individual(s) for the publication of any potentially identifiable images or data included in this article.

## Author Contributions

BE and YB: had full access to all data in the study and take responsibility of the integrity of the data and the accuracy of the data analysis, study concept and design, drafting of the manuscript, and study supervision. BE, ZT, and YB: acquisition, analysis, or interpretation of data. BE, ZT, KF, and YB: critical revision of the manuscript for intellectual content. ZT: statistical analysis. BE: obtained funding.

### Conflict of Interest

The authors declare that the research was conducted in the absence of any commercial or financial relationships that could be construed as a potential conflict of interest.
